# Transactions of Kings County Medical Society

**Published:** 1864-11

**Authors:** 


### TRANSACTIONS OF KINGS COUNTY MEDICAL SOCIETY.

We take great pleasure in announcing to our readers that the Kings County Medical Society have resumed the publication of their transactions which was suspended by the suspension of the *American Medical Monthly* in 1862. This is one of the most vigorous, capable and active medical associations in this country, and furnishes annually many very valuable papers, upon medical and scientific subjects. These will hereafter appear regularly in the original department of our Journal, and their volume of transactions will be published from it. In our next issue will be published a paper upon *Tracheotomy in Diphtheria*, with an abstract of the discussion which followed. This paper was sent as a contribution from Kings County Society to the New York State Medical Society, and was published in their Transactions. By vote the publication of their Transactions includes such papers as have been published elsewhere during the period of suspension, which it is desirable to have re produced. Notwithstanding this, it will all be published connectedly as strictly original matter. It is new and original, though a few of the papers have been isolated from the discussions and previously published. We should take it as highly complimentary to receive for publication the Kings County Society, only we discover upon reflection that the *Buffalo Medical and Surgical Journal* is the only monthly medical periodical now published in the State of New York, indeed we may say the only medical journal, except the foreign reprints, and on this account mainly we suppose the award of this favor is based. We are highly gratified with the opportunity to present our readers with these papers and the discussions thereon. We have no doubt it will prove interesting and instructive in the highest degree.

